# Intraoral Ultrasonography for Periodontal Tissue Exploration: A Review

**DOI:** 10.3390/diagnostics13030365

**Published:** 2023-01-18

**Authors:** Matthieu Renaud, Alexis Delpierre, Hervé Becquet, Rachid Mahalli, Guillaume Savard, Pierre Micheneau, Delphine Carayon, Frederic Denis

**Affiliations:** 1 Faculty of Dentistry, Tours University, 37000 Tours, France; 2 EA 42-03 Biosanté et Nanoscience, Faculty of Dentistry, Université Montpellier, 34000 Montpellier, France; 3 Department of Odontology, Tours University Hospital, 37261 Tours, France; 4 Department of Odontology, Montpellier University Hospital, 34000 Montpellier, France; 5 EA 75-05 Education, Ethique, Santé, Faculté de Médecine, Université François-Rabelais, 37000 Tours, France

**Keywords:** ultrasonography, periodontal tissue, periodontology, high frequency ultrasound imaging, periodontal imaging

## Abstract

This systematic review aims to investigate the possibilities of ultrasound imaging in the field of periodontal tissues exploration to visualize periodontal anatomical structures and to assess reliability in clinical evaluation using the PRISMA guidelines. An electronic search through the MEDLINE database was realized to identify studies that have explored ultrasonography in the field of periodontal imaging published from 2000 to March 2022. The search resulted in 245 records; after exclusions, a total of 15 papers were included in the present review. Various publications have shown the possibility of using intraoral ultrasound for a precise exploration of intraoral tissues and to perform measurements of periodontal structures. Studies argue that ultrasounds open the prospect of a complete paradigm shift on the diagnosis and follow-up of periodontal disease. However, there is currently no clinical device dedicated to periodontal ultrasound. This field is still under-studied, and studies are needed to explore the large field of applications from periodontal assessment to treatment reassessment, including surgery. Researchers should focus their efforts to develop special intraoral ultrasound device and explore the possibilities of clinical periodontal applications.

## 1. Introduction

Periodontitis is an inflammatory disease of the periodontium (tooth supporting tissues) of bacterial origin. It can be treated and controlled successfully, to avoid the progressive destruction of the tissues (the gum, the periodontal ligament and the alveolar bone) [1]. A patient with periodontitis treated and stabilized with initial treatment nevertheless presents a risk of recurrence. Therefore, a continuous and individual assessment of the patient’s risks is necessary in order to monitor the non-evolution of the pathology [2]. Clinically, gingival health is defined by the absence of bleeding on probing, erythema and edema, patient symptoms, attachment and bone loss [3]. The bleeding on probing test performed by a periodontal probe into the bottom of the sulcus is one element of monitoring the health or inflammation of the gingival tissues that is best documented in the literature [4]. This test is important because it has been shown that bleeding is an earlier sign of gingivitis than are the visual signs of inflammation [5]. Bleeding on probing is an indicator of pathological phenomenon with presence of pocketing. On the other hand, it has been demonstrated that bleeding on probing provoked with pressures greater than 0.25 Newtons (N) results in false-positive readings in lack of pathology [6]. Despite this, periodontal probing has its limitations. This test is time-consuming, and operator-dependent errors are possible, such as incorrect angulation of the probe, excessive probing pressure on the gingival tissues, and incorrect reading of the measurement on the probe. An error in calculating the loss of attachment may occur. Reading errors can also result from interference from calculus, the presence of an overhanging restoration, or the contour of the crown. Other factors, such as the size of the probe tip, angle of probe insertion, accuracy of probe calibration, and degree of inflammation in the periodontal tissues affect sensitivity and the reproducibility of the measurements [7].

Radiographic evaluation is an essential element of periodontal diagnosis. A radiograph of periodontal health includes a normal lamina dura, presence of bone in furcation areas, and 2 mm distance from the most coronal portion of the alveolar bone crest to the cementoenamel junction of the tooth [8]. Alveolar bone loss has the result of bone resorption in response of inflammatory process during periodontitis development. That is why it is very difficult to evaluate clinical periodontal health only using routine radiographs [3].

In view of these limitations, it is important to consider a new type of exploration with less time-consuming and easier to use in clinical practice. Since the middle of 1980s, several publications have presented ultrasound imaging applied to the exploration of the oral cavity in animals [9]. Ultrasonographic of the periodontal tissues could constitute an alternative for the visualization of the periodontal structures and for the detection of periodontal disease with the detection of periodontal pocket and the presence of deep tissue inflammation.

Ultrasonography is an ultrasonic medical imaging technique used in a large field of medicine. The ultrasounds used in medical ultrasonography are mechanical waves which propagate in a medium. A mechanical wave is a local deformation which is propagated step by step in solid, liquid or gas medium [10,11]. The frequency of the wave corresponds to the number of periods per second in Hertz (Hz). The field of ultrasound is between 20 kHz and 200 MHz [12]. In medical imaging, ultrasound scanners from 1 to 15 MHz are used. High frequency ultrasound is defined above 20 MHz [13]. This non-ionizing technique allows soft tissues exploration of the human body through ultrasonic waves, which makes it possible to have resolutions between 0.4 and 2 mm. High frequency techniques were used for medical applications such as obstetric, cardiology, abdominal and muscular explorations [14,15,16]. Optimization in B-mode ultrasound has permitted to achieve the best possible image quality in medical applications and develop a wild field of applications [17]. To obtain resolutions better than 0.1 mm, it is necessary to use ultrasound frequencies above 20 MHz. This field of ultra-high resolution ultrasound imaging has permitted the exploration of the skin [18] and the eye [19] for small animal imaging or angiology by submillimeter resolution of small structures [16,20].

Ultrasound imaging has the potential to complement routine radiographic imaging in periodontology and provides instantaneous images of anatomical structures during the same examination. The practitioner can directly modify the incidence of the probe according to the tissue anatomy. It can be used without risk in all patients because it is not ionizing. Overcoming the phenomenon of superposition is possible with this type of imaging, unlike with 2D imaging [21]. For application in oral and dental imaging, its qualities depend on its ability to accurately capture these complex structures in a simple and rapid manner. These complementary methods are attractive because they are non-irradiating, non-invasive and comfortable for the patient since they allow direct reading of the images.

In these perspectives, studies showed the validity and reliability of ultrasonography in the measurement not only of gingival thickness but also of other periodontal structures which cannot be assessed through inspection and palpation [22,23]. A 25 MHz high-frequency resolution ultrasound probe, specially designed for intraoral applications, provides additional morphological information that is not accessible by conventional dental X-rays in daily dental practice with a large-scale of application in the diagnosis of pathologies [24].

Therefore, the aim of this systematic review was to investigate the possibilities of ultrasound imaging in the exploration of periodontal tissues to visualize periodontal anatomical structures and to assess reliability in clinical evaluation.

## 2. Materials and Methods

This systematic review was reported according to the PRISMA guidelines for Systematic Reviews [25].

### 2.1. Search Strategy

An electronic search was conducted through the MEDLINE (PubMed) database to identify publications that met the inclusion criteria. (9) The search was performed from 2000 up to March 2022, in order to identify the studies that explore the contribution of ultrasound imaging in periodontology, using the following search terms and keywords alone or in combination with the Boolean operator “AND”/“OR” according to the following equation (“alveolar ridge” [Mesh]) OR (“alveolar bone” [Mesh]) OR (“caries” [Mesh]) OR (“cementoenamel junction” [Mesh]) OR (“periodontal attachment” [Mesh]) OR (“periodontal probing” [Mesh]) OR (“periodontal charting” [Mesh]) OR (“dental implant” [Mesh]) OR (“periodontitis” [Mesh]) OR (“gingivitis” [Mesh]) OR (“periodontium” [Mesh]) AND (“sonography” [Mesh]) OR (“diagnostic ultrasound” [Mesh]) OR (“ultrasonography” [Mesh]).

### 2.2. Study Detection

References of the eligible studies on the topic were manually checked, and two independent operators (F.D. and M.R.) screened the studies according to the inclusion/exclusion criteria. In case of disagreement, a 3rd reviewer (R.M.) was asked.

#### Inclusion and Exclusion Criteria

We included experimental or clinical studies (longitudinal, cross-sectional or randomized studies), in healthy patients or patients with periodontitis, that explored the link between ultrasound imaging and periodontology or oral tissues or explored an association between ultrasonography and histological or histometric characterization of periodontal tissues. According to the type of ultrasound imaging, we included only studies that presented a mode B ultrasound device. We excluded conferences, abstracts, reviews and editorials. Publications concerning the detection of carious lesions and publications relating the utilization of ultrasound to increase the healing and osseointegration potential of dental implants were not included, and we excluded publications related to other medical disciplines.

Each study that met the inclusion criteria was analyzed from several aspects such as authors, date of publication, study design, ultrasound device description, image classification, results, limitations and discussion.

### 2.3. Data Collection

The list of titles and abstracts to identify the potentially relevant papers based on the inclusion criteria previously announced were independently screened by two reviewers (F.D. and R.M.). If the abstracts were identified as non-relevant, the full studies were reviewed to decide if they should be included or not according to the inclusion criteria. A scan of the references of the previously selected articles completed the selection to improve the systematic review. When a discrepancy in the selection decision appeared, the two reviewers engaged in discussion until a consensus was found. If needed, a third reviewer (M.R.) resolved the possible conflicts concerning eligibility.

## 3. Results

### 3.1. Study Selection

**Figure 1:**
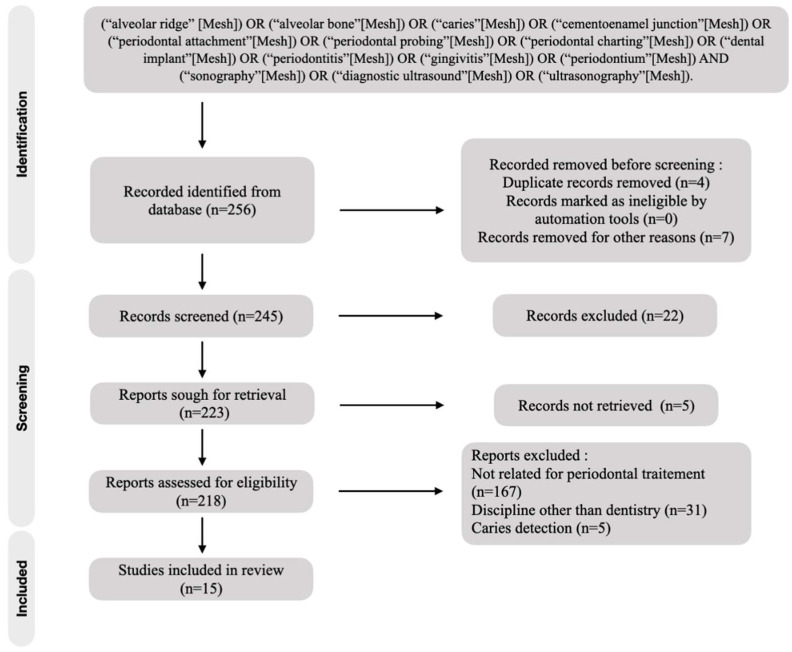
Flow chart of included studies.

The initial studies retrieved from the databases were first selected, and studies that met the eligibility criteria were reviewed and analyzed. After 220 reading abstracts and 7 full articles, only 15 articles were selected from the 245 studies. The percentage of agreement between the reviewers was 100%. The complementary detection did not result in the selection of new publication for analysis. Finally, a total of 15 articles were included in the analysis (Figure 1).

Among the 15 studies included in the analysis (Table 1), we were able to distinguish four common themes emerging such as the evolution of trials, ultrasound device presentation, the description of periodontal tissues using ultrasonography and comparative ultrasonography measurement. Studies included in the analysis were in vitro or ex vivo studies or clinical comparative trials using different tools to validate the use of ultrasound devices. Most of them applied low ultrasound frequencies.

### 3.2. Test Evolution

**Figure 2:**
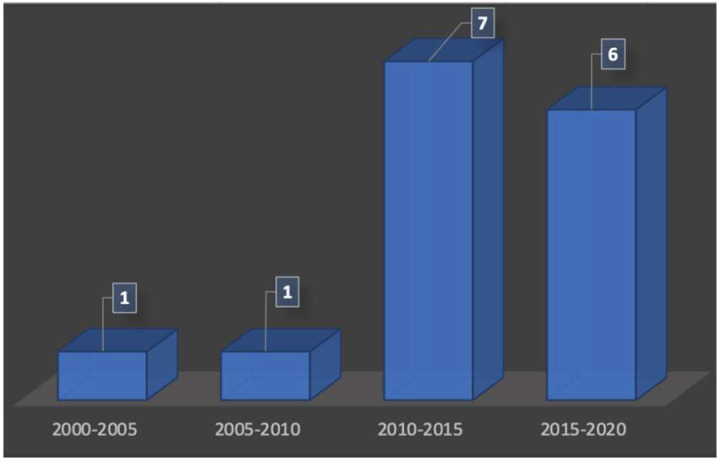
Number of publications over the time.

Using ultrasonography for the visualization of the structures of the oral cavity was not an actual possibility, and publications existed for many years already. According to the periods of publications used in this review, we have noticed that there is a renewed interest in ultrasound technologies for periodontal exploration from the 2010s (Figure 2). There were only two publications between the years 2000 and 2010, whereas the number of publications increased after 2010 with 13 articles found.

According to our analysis, researchers have set up feasibility trials [23,33] for imaging and measuring the periodontal tissue. On the other hand, they have subsequently set up pilot trials [24,37] to evaluate the measurements provided by ultrasound images. In only one publication [29], ultrasonography is used to assess the size of the gingival tissue before and after professional periodontal cleaning.

**Figure 3:**
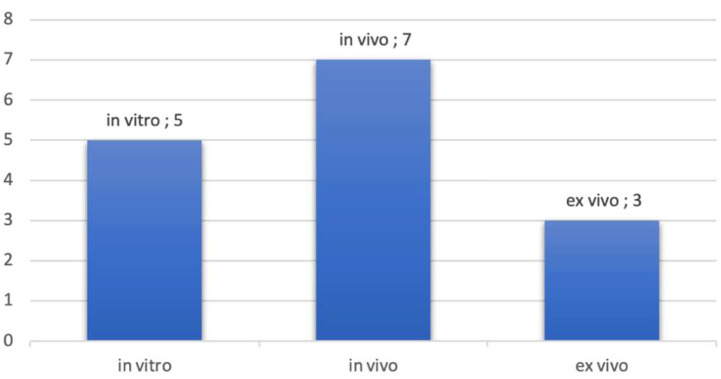
Number of publications by type of exploration.

Most of the studies were in vivo studies. In vitro studies used pig jawbones while ex vivo studies were performed on cadavers. Figure 3 presents the proportion of studies according to the nature of the exploration. It should be noted that some studies have been realized in vitro and in vivo topic. This is the case, for example, in the studies of Sun et al., where measurements were realized on pig jawbones and on patients directly [36].

### 3.3. Overview of Ultrasonographic Devices

The literature presents a set of ultrasound probes used for the majority with an intraoral approach. The probe is in contact directly with intra oral tissue for image production providing a sagittal slice. In two publications [28,33], we have found ultrasound probes for extra-oral approach. For the most part of them, ultrasound probes were not used for intra-oral utilization. In some studies, ultrasonography was used for monitoring skin [26,30] or used in small animals’ studies [34].

In the study by Salmon et al., [24] the probe has been designed for intraoral use. It was manufactured on the model of a handpiece to use on all surfaces of the tooth. Tattan et al., [37] as well as Chan et al., [32,33] also have used probes designed for intraoral use. The prototypes for the oral cavity are smaller and used a head probe with a special angle allowing the tightest spaces of the oral cavity access. Components such as the transducer are necessarily miniaturized [24] for ease of use. Coupling agent is often a commercial coupling gel which is similar to water. The coupling gels used were not specific to the oral environment or to use in periodontology.

Regarding the acoustic parameters of the probes, it should be remembered that periodontal imaging aims to image structures close to the probe head. The depth of exploration is less than 10 mm, and the structures to be explored are sub-millimetric in size. In this specific case, emission of frequencies needs to be between 15 MHz [33] up to 40 MHz [23,28] for best results. Beyond 20 MHz, we enter in the field of high frequency ultrasound to obtain high resolution images, with a resolution of less than 100 microns [24,34,35]. The images obtained can be modified to allow better readability as with X-ray images. For example, in the study by Chifor et al. [30] the authors have implemented an image processing technique that automatically delimits the sulcus successfully.

### 3.4. Periodontal Tissues Description with Ultrasonographic Imagery

**Figure 4:**
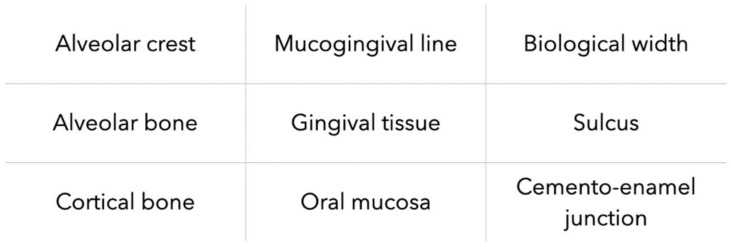
Types of periodontal tissues imaged by US imaging.

Within the periodontal tissue, bone structures are more echogenic because they reflect more ultrasound waves. Impedance rupture between hard and soft tissues allows them to be clearly distinguished. Therefore, hard tissues appeared whiter than soft tissues on the gray scale. First images need to be processed by computer. The authors added different colors according to the tissues in order to allow a good tissue differentiation. Periodontal tissues images from more recent publications, such as that of Tattan et al. [37] show better resolution with an adapted gray scale. Analysis of selected articles showed that ultrasound provided images of all periodontal structures, in pigs and humans, as listed in Figure 4.

### 3.5. Types of Measurements and Comparison

Detection and classification of periodontal disease is based on measurements of periodontal tissue. The studies almost systematically present a comparison between the ultrasound measurements of the periodontal tissue and the measurements obtained by procedures used in clinical routine (clinical probing), qualified as the gold standard. Publications show measurements of the sulcular depth [23], the thickness of the free gingiva [23], the thickness of the attached gingiva [26,28], the biological width [23,26,28], the level of the alveolar crest in relation to the cemento-enamel junction [21,27,28,32,36], the thickness of the cortical bone [26,32], the height of the interdental papilla [37] and the gingival thickness on the edentulous ridge [37].

Studies on porcine jaws show direct transgingival measurements using an endodontic file [21,34,36], direct histological measurements [21] and direct clinical measurements using periodontal probe [21,23,28,32,34,37]. Conventional imaging methods have also been used: cone been computerized tomography (CBCT) [27,31,32,37], retro-alveolar radiography [28] and optical microscopy [27,30,35].

Several authors have used statistical tools to establish the correlation between ultrasound measurements and other means of measurement [27,28,30,32,36,37]. In the work of Zimbran et al. [23] the measurement of the sulcular space using the periodontal probe (gold standard) was not statistically different (*p* < 0.05) compared to the ultrasound measurement. The greatest measurement variation between the two means of measurement did not exceed 0.5 mm. Tsiolis et al. [21] have calculated the repeatability coefficient for the ultrasound measurements in comparison with in vitro measurement. Ultrasound measurements were better. Chifor et al. [29] highlighted the reproducible nature of ultrasound measurements. The intra-observer ICC calculated for ultrasound measurements was 98.8 with *p* < 0.001 for the measurement of the distance between the cemento-enamel junction and the alveolar ridge. After publications analysis, ultrasonography demonstrates reproducibility and precision. Ultrasonography appeared reliable in comparison with to other means of measurement (clinical and radiographic) in periodontal tissue application.

## 4. Discussion

Ultrasound for periodontology has benefited from renewed interest since the 2010s. However, there are only few teams working on this topic. The teams that presented several publications were made up of researchers from different research centers with different levels of expertise. The team of Chan et al. [32,33] was made up of professionals skilled in medical imaging and dental surgery, like the study by Tattan et al. [37] which involved both South Korean and American practitioners. Moreover, this review exposed in vitro studies that allowed direct measurements only possible in animals, such as transgingival measurements and histological samples. Tattan et al. [37], in the context of measuring gingival thickness on an edentulous ridge, were able to verify their results by direct measurements on humans. Only one publication [29] presented measurements on a pathological periodontium before and after a treatment session using ultrasonography. Patients recruitment for future studies should include patients requiring periodontal surgery to benefit from direct measurement of periodontal tissues. The results of the ultrasonography measurements are encouraging. Due to the diversity of the in vitro and in vivo protocols and the devices used, it was not possible to perform a meta-analysis.

Ultrasound imagery produces artifacts that are unique such as the “drop shadow”. This is due to the fact that a structure always appears less echogenic (blacker) than it really is when it is preceded (on the path of the ultrasound) by a hyperechoic structure. The study by Salmon et al. [24] reported the presence of artifacts due to non-specific coupling gel and distortions on ultrasound images. Ultrasound imagery offers a systematic buccal-palatal section of the tooth and the periodontal tissues. This reconstruction view is incomplete, as it does not allow the entire tooth to be imaged. To be precise, the ultrasound probe must be systematically passed over the vestibular and palatal surfaces to image the globality of the tissues around tooth. Most ultrasonography devices are borrowed from other medical disciplines, and for those that are not, they are prototypes. In addition to the evaluation of the ultrasound images, their handling and ergonomics were evaluated. Most studies show that the tool must be handy and small enough for use in the mouth. The miniaturization of the ultrasound system must also allow a high definition of the image which is a real challenge in terms of technological development. The articles on the PubMed database are mainly addressed to the medical community. However, the description of the acquisition protocol of the ultrasound imaging is not detailed or sometimes not presented. In the context of this research, none of the publications clearly mentioned the participation of a commercial partner or the cost/effectiveness ratio of ultrasound imaging compared to existing means. Technological locks persist and require the continuation of research funding. According to dental access, to produce new probe heads suitable for perfecting image processing software and continuing the process of miniaturization of the transducer, it is necessary to improve the utilization of the imagery approach. Dentists are not trained in reading ultrasound images, and the results obtained with an ultrasonography device must be clear and easy to read and to analyze.

The patients recruited for the studies were mostly healthy patients. Only Chifor et al. [29] enrolled patients with gingivitis. We can legitimately assume that the scanning techniques will have to adapt in the event of a bone defect or in the event of the presence of deep pockets (more than 6 mm). In addition, clinical measures such as periodontal probing are estimated to within half a millimeter, while the ultrasound technic proposes a measure less than tenths of a millimeter. There is a difference in scale between the interpretability of the measurements that can be obtained in the clinic and the precision of the ultrasonography. However, this literature review showed the accuracy of ultrasound for measurements at the level of anatomical structures. [35] Ultrasonic imaging can be valuable for accurate and real-time periodontal diagnosis without concerns about ionizing radiation [35,37]. The possibility to use ultrasound for minimally invasive surgery is founded [32]. In the future, the new prototypes should aim for a scanning time of 5 min for the entire oral cavity, compared to less than one minute per tooth today [37] with an intraoral approach. To do this, dental surgeons must appropriate this technology to be more efficient in the detection of the periodontal diseases.

## 5. Limitations

This work should not give rise to hasty conclusions due to the low number of publications and the intrinsic limits of the studies. Indeed, the use of only one electronic database is a particular limitation of this study. On the other hand, there are many biases in these studies and those related to the operators’ manipulations. In addition, many of the studies presented are animal studies that do not fully correspond to clinical conditions. These are feasibility studies and pilot tests with a medium level of proof (grade B). In addition, the heterogeneity of frequencies and acquisition protocols used in the current literature may hinder direct comparison between studies and necessitate more studies with homogeneous ultrasound measurements. All of the studies presented here do not allow to draw conclusions on the routine clinical use of ultrasounds in periodontology and require the setting up of randomized clinical studies.

To improve our analysis of the literature, certain sources of complementary information from other fields of science are necessary. For example, there are specific databases for biotechnology engineers and acousticians. We can find technical information that would consolidate the state of the art. The articles exclusively dedicated to implantology also present US technologies allowing indirect exploration of periodontal tissues.

## 6. Perspectives

High-resolution images have an equal or greater degree of precision than conventional imagery. It is a non-ionizing imaging that overcomes the discomfort of periodontal probing for diagnosis. Ultrasound pocket depth measurements appear to be as reliable and reproducible as those obtained by manual probing, even with an electronic probe.

The ultrasound system combined with an artificial intelligence (AI) data processing system opens the way to new perspectives regarding the distinction of the sulcular space and the measurement between the cemento-enamel junction and the sulcus. On this type of standardized measurements, the process could be automatized, and the data integrated into periodontal charting. We would then have a reliable, less time-consuming device involved in patient follow-up. The AI will allow a better handling of the device.

The use of ultrasound can be beneficial at all times of periodontal surgery. The ultrasonography can be allowed to see the quality of the graft directly before surgery. They would allow the incision path to be refined to avoid vasculo-nervous structures. Postoperatively, the healing or not of the graft could be monitored by inspecting the interactions with the recipient bed. Finally, inter-site contamination that may be caused by manual probing could be avoided.

## 7. Conclusions

Interest in intraoral ultrasound technologies has grown over the past decade. The various publications highlighted a reliable means of imaging allowing a precise exploration of the periodontal tissues offering the possibility of carrying out measurements of the periodontal structures themselves or between them. However, there is currently no clinical device dedicated to periodontal ultrasound. For the moment, the use of ultrasound in periodontology remains confined to the field of research. Ultrasound opens the prospect of a complete paradigm shift on the diagnosis and follow-up of periodontal disease, by reducing the examination time of periodontal pockets, being more reproducible and more efficient. In addition, the implementation of software including an artificial intelligence system allowing the direct measurement of the periodontal pocket and the early detection of the inflammation of the deep periodontium could permit easy diagnosis and early treatment of periodontal disease. Moreover, this new approach could allow an evaluation of the initial treatment and permit more reliable periodontal maintenance. In addition, the direct measurement of periodontal pockets without ionizing radiation is a major advance on the complementary examination allowing to reduce the number of periapical X-rays. Periodontal ultrasound is not intended to replace the conventional means of evaluation of periodontal tissues. However, the associated opportunities are immense with a lot of applications from periodontal assessment to treatment reassessment, including surgery.

## Figures and Tables

**Table 1:** Data analysis.

Reference	Study Design	Study Quality	Objectives	Frequency of Ultrasound Devices	Nature of Tissues	Comparison	Main Results and Limitations
Tsiolis et al., 2003 [21]	In vitro study. N = 4, 3 posterior teeth per pig jaw, measurement repeated 2 times	Grade B*	To study high-frequency ultrasound imaging for periodontal examination	f = 20 MHz	Alveolar crest and gingival tissue	Transgingival probing, direct probing, histological comparison	The periodontal structures could be imaged. Measurements with ultrasound are precise and reproducible: less than 0.5 mm difference for all measurements with ultrasound compared to direct measurements. Possible distortion of ultrasound images providing cuts according to an Incomplete buccal-lingual section. Its need to work to have the soft tissues and the hard tissues distinctly.
Chifor et al., 2010 [26]	In vitro study. N = 4, 20 teeth. Measurement of the lingual mandibular part	Grade B*	Establishment of an experimental model to establish whether high-frequency ultrasound can provide relevant information about the periodontal tissues	f = 20 MHz	Alveolar and cortical bone, root surface, periodontal space width, gingival tissues	Literature regarding dento-periodontal anatomy in the domestic pig	Measurement with an accuracy of one hundredth of a millimeter. Ultrasound can be an alternative to conventional imaging. Need for a miniaturized transducer.
Chifor et al., 2011 [27]	In vitro study. N = 4, 20 teeth. Measurement of the lingual mandibular part	Grade B*	Identify by ultrasonography the reference points necessary to monitor the horizontal bone resorption and to assess the accuracy of the measurements	f = 20 MHz	Cemento-enamel junction, root surface, periodontal space width, alveolar and cortical bone	CBCT, direct microscopy section	Strong parametric correlation between microscopy versus US *p* < 0.001. Linear regression shows a statistically significant correlation between CBCT measurements and microscopy for alveolar bone *p* < 0.05. Usefulness in the screening and monitoring of bone resorptions.
Salmon et al., 2012 [24]	Clinical study. N = 3, teeth were explored on the lingual and buccal sides (162 samples) by two independent radiologists	Grade B*	Present clinical intraoral ultrasound images interpretable, to identify the relevant applications of this novel tool and to design future oral studies. Test the ergonomics of the device.	f = 25 MHz	Tooth surface, alveolar bone, gingival epithelium, sulcular space, mucogingival line, oral mucosa	NC	Periodontal tissues are visible in more than 90% of cases. The ergonomics of this device is adapted and allows a fast and comfortable measurement.
Zimbran et al., 2013 [23]	Clinical study. N = 4, 4 posteriors teeth of the lower jaw were imaged from buccal incidence	Grade B*	To investigate the possibility to use high-frequency ultrasound imaging for the assessment of periodontal tissues	f = 40 MHz	Alveolar bone, crown tooth, sulcular space, free gum	Clinical measure: periodontal probing, clinical crown measurement	No statistical difference between clinical measurements and ultrasound measurements
Chifor et al., 2015 [28]	Clinical study. N = 10, 49 teeth with marginal periodontal disease, on upper and lower pre-molars and frontal teeth, 245 measurements.	Grade B*	Identify the information for diagnosis and staging of periodontal disease using ultrasonography.	f = 40 MHz	Alveolar crest, cemento-enamel crest, free gum, root surface/gum recession, sulcular space	Clinical examination (periodontal probing) and digital periapical X-rays	All structures could be accurately imaged. Very good correlation on all US measurements versus clinical measurements with student test and correlation coefficient R. Scan time decreases as operator learns.
Chifor et al., 2015 [29]	Clinical study. N = 18, teeth without restorations having subgingival calculus (upper and lower premolars and frontal teeth). Measurements realized before and after periodontal treatment (J + 2 and J + 7), gingival Index and Sulcus Bleeding Index calculated.	Grade B*	To evaluate the usefulness of periodontal ultrasonography in the assessment of gingival inflammation, following professional teeth cleaning.	f = 40 MHz	Alveolar and cortical bone, enamel, cementum, cemento-enamel junction, keratinized epithelium	NC	Ultrasound measurements are accurate and repeatable (*p* < 0.001). Usefulness in the measurement of bone resorption and gingival inflammation. Non-invasive and efficient method requiring further studies with control groups. Requires new transducers adapted to the oral cavity.
Chifor et al., 2015 [30]	In vitro study. N = 8, 36 sites on lingual surfaces	Grade B*	To demonstrate that periodontal ultrasonography is a reliable method with which to identify and evaluate the attachment level of the gingival junctional epithelium.	f = 20 MHz	Enamel, root surface, sulcular space, free gum, alveolar and cortical bone	Direct microscopy	Variation between processed images and microscopy measurements between 0.06 and 1.75. Need for more clinical studies. Transducer size should be reduced.
Nguyen et al., 2016 [31]	In vitro study. N = 4, 2 porcine lower incisors, 6 measures per tooth.	Grade B*	To measure periodontal soft and hard tissues	f = 20 MHz	Enamel, dentine, dentin-pulp junction, cemento-enamel junction, free and attached gum, alveolar and cortical bone	CBCT	All the structures could be precisely identified with a difference of less than 10% between ultrasound versus CBCT measurements. R < 0.5 mm. The probe head is small enough to consider clinical trials on all dental sites.
Chan et al., 2017 [32]	Ex vivo study. N = 6, dental and periodontal tissues at the mid-facial site of each tooth on fresh cadavers (144 teeth in total)	Grade B*	To evaluate the accuracy of using ultrasound to measure facial crestal bone level and thickness	f = 14 MHz	Alveolar crest, cemento-enamel junction	CBCT, direct measurements	Correlation coefficient r > 0.80 for CBCT versus ultrasound and ultrasound versus direct measurements. Difference of measurement means: <0.1 mm.
Chan et al., 2017 [33]	Ex vivo study. N = 1, multiple measurements relevant to the facial bone surface and soft tissue of maxillary anterior teeth, the greater palatine foramen, the mental foramen, and the lingual nerve.	Grade B*	To investigate ultrasound to image soft tissue, hard tissue surface topography and specific vital structures.	f = 14 MHz	Distance alveolar edge–cemento-enamel junction, oral mucosal thickness, greater palatine foramen diameter and associated mucosal thickness, mental foramen diameter, lingual nerve diameter	CBCT, direct measurements, anatomical dissection	Enamel, cement-enamel junction, root surface, alveolar bone surface visible with cone of shadow behind these structures. Requires clinical studies with a larger sample. Possibility of using ultrasound for minimally invasive surgery.
Lin et al., 2017 [34]	In vitro study. 39 porcine teeth (12 teeth with artificially deeper pockets), 4 measures per tooth, i.e., 156 measures.	Grade B*	To investigate photoacoustic ultrasound for high–spatial resolution imaging of probing depths. Specific contrast from dental pockets was achieved with food-grade cuttlefish ink as a contrast medium.	f = 21 MHz or 40 MHz	Marginal gingiva, alveolar bone, root surface	Periodontal probing, endodontic file	Statistically significant differences between the 2 measurement approaches. The photoacoustic imaging approach also offered 0.01-mm precision and could cover the entire pocket, as opposed to the probe-based approach, which is limited to only a few sites.
Barootchi et al., 2020 [35]	Ex vivo study. N = 9, 3 areas in the lingual mandible (premolar, molar and retromolar). N = 19, 1 measure per patient (lingual nerve)	Grade B*	To validate ultrasound in measuring the mandibular lingual structures on human cadavers and to test its feasibility in imaging the lingual nerve in live humans	NC	Lingual mucosa, mylohyoid muscle, lingual nerve	Histological section	R correspondence (*p* < 0.05) between histological and ultrasound measurements. First publication demonstrating the accuracy of ultrasound for measurements at the level of anatomical structures.
Sun et al., 2020 [36]	In vitro study. N = 4, 6 measurements per teeth. And clinical study. N = 50, 400 premolars with healthy periodontium	Grade B*	To measure the buccal gingival thickness and alveolar crest thickness of premolars using ultrasonography and to explore the relationship between gingival thickness and alveolar crest thickness	f = 15 MHz	Alveolar bone, alveolar crest, cemento-enamel junction, gingival tissue	Periodontal probing, endodontic file	Strong correlation between ultrasound measurements (r > 0.8) and with the file on pork jawbone (*p* < 0.05). The gingiva at the level of the maxillary premolars and the alveolar ridge thickness are greater in men compared to women (*p* < 0.05). Limits: recruitment with a reduced age group with a sample that will have to be larger
Tattan et al., 2020 [37]	Clinical study. N = 20, 40 teeth and 20 single missing tooth gaps from 20 patients scheduled to receive a dental implant surgery	Grade B*	To evaluate the correlation and accuracy of ultrasound in measuring periodontal dimensions, compared to direct clinical and cone-beam computed tomography methods	f = 24 MHz	Interdental papilla, gingival tissue, oral gum, alveolar crest	CBCT, periodontal probing	The mean difference in mucosal thickness between the ultrasound and direct readings was −0.015 mm (95% CI: −0.655 to 0.624 mm) without statistical significance. ICC between ultrasound and CBCT ranged from 0.654 to 0.849 among the measured parameters. The mean differences between ultrasound and CBCT range from −0.213 to 0.455 mm, without statistics significance. Ultrasonic imaging can be valuable for accurate and real-time periodontal diagnosis without concerns about ionizing radiation.

* Grade B: Grade of recommendation, quality of evidence, moderate [38].

## Data Availability

Not applicable.
